# Chemical Cues from Entomopathogenic Nematodes Vary Across Three Species with Different Foraging Strategies, Triggering Different Behavioral Responses in Prey and Competitors

**DOI:** 10.1007/s10886-021-01304-8

**Published:** 2021-08-20

**Authors:** John M. Grunseich, Natalie M. Aguirre, Morgan N. Thompson, Jared G. Ali, Anjel M. Helms

**Affiliations:** 1 Department of Entomology, Texas A&M University, College Station, TX 77843 USA; 2 Department of Entomology, The Pennsylvania State University, University Park, PA 16802 USA

**Keywords:** Belowground chemical ecology, Context dependence, Non-consumptive effects, Predator hunting mode, Predator cues, Predator–prey interactions

## Abstract

Chemical cues play important roles in predator–prey interactions. Semiochemicals can aid predator foraging and alert prey organisms to the presence of predators. Previous work suggests that predator traits differentially influence prey behavior, however, empirical data on how prey organisms respond to chemical cues from predator species with different hunting strategies, and how foraging predators react to cues from potential competitors, is lacking. Furthermore, most research in this area has focused on aquatic and aboveground terrestrial systems, while interactions among belowground, soiling-dwelling organisms have received relatively little attention. Here, we assessed how chemical cues from three species of entomopathogenic nematodes (EPNs), each with a different foraging strategy, influenced herbivore (cucumber beetle) and natural enemy (EPN) foraging behavior. We predicted these cues could serve as chemical indicators of increased predation risk, prey availability, or competition. Our findings revealed that foraging cucumber beetle larvae avoided chemical cues from *Heterorhabditis bacteriophora* (active-foraging cruiser EPNs), but not *Steinernema carpocapsae* (ambusher EPNs) or *Steinernema riobrave* (intermediate-foraging EPNs). In contrast, foraging *H. bacteriophora* EPNs were attracted to cues produced by the two *Steinernema* species but not conspecific cues. Notably, the three EPN species produced distinct blends of olfactory cues, with only a few semi-conserved compounds across species. These results indicate that a belowground insect herbivore responds differently to chemical cues from different EPN species, with some EPN species avoiding prey detection. Moreover, the active-hunting EPNs were attracted to heterospecific cues, suggesting these cues indicate a greater probability of available prey, rather than strong interspecific competition.

**Supplementary Information:**

The online version contains supplementary material available at 10.1007/s10886-021-01304-8.

## Introduction

A major goal among ecologists is to better predict the outcomes of trophic interactions and their cascading consequences for community ecology and ecosystem function (Miller et al. 2014; Culshaw-Maurer et al. 2020; Descombes et al. 2020). Growing evidence in the study of predator–prey interactions points to environmental (e.g., climate and habitat) and species (e.g., predator and prey) traits as playing key roles in disentangling this complexity (Rosenheim et al. 2004; Luttbeg et al. 2020; Wirsing et al. 2021). Behavioral traits of both predators and prey are of increasing interest, particularly the role these traits play in non-consumptive effects. Non-consumptive effects—in contrast to ‘consumptive effects’, which describe the capture and killing of prey by predators—encompass modified prey behavior, morphology, and/or physiology in response to perceived predation risk (Thaler et al. 2012; Hermann and Landis 2017). For instance, prey may reduce foraging activity or escape to different habitats to circumvent predators (Heithaus et al. 2009; Hermann and Thaler 2014), highlighting the challenge prey face in evading predation while also locating suitable food resources (Sih 1980). Predators also face foraging challenges as they compete with other predators for prey, without falling victim to predation themselves (Rosenheim 1998). To forage for prey, predators employ different hunting behaviors or modes. Some predators are active hunters that move through the environment to locate and pursue prey, while others adopt a sit-and-wait or ambush strategy, remaining stationary and attacking prey that move within close range (Schmitz 2008; Miller et al. 2014). Current theory predicts prey should most readily detect and respond to cues from ambush predators that represent an immediate threat, possibly because these cues are more highly concentrated. In contrast, cues from active predators are likely more diffuse and less reliable information sources, as prey must balance the cost of anti-predator vigilance and reduced foraging against the lower likelihood of encountering these active predators (Kats and Dill 1998; Preisser et al. 2007). Theory also predicts predators should avoid cues from potential competitors, particularly those that will outcompete or predate them (Rosenheim 1998; Chase et al. 2002; Mestre et al. 2020). Here we test these predictions by 1) examining prey responses to chemical cues from three species of entomopathogenic nematodes (EPNs) with different foraging strategies and 2) evaluating how these cues affect the foraging behavior of an active-hunting EPN species.

Trophic interactions are often mediated by chemical information, which provides a mechanistic link to observed behaviors. It has been well documented, for example, that insect herbivores use plant-produced chemical cues to select suitable hosts, while their natural enemies typically rely on herbivore-associated cues to locate prey (Bruce and Pickett 2011; Pearse et al. 2020; Grunseich et al. 2020a). Many species (e.g., plants and herbivores) have evolved to recognize chemical cues associated with their enemies to help them predict and avoid attack (Helms et al., 2017; Hermann & Thaler, 2014; Kats & Dill, 1998; Kempraj et al., 2020; Karban et al. 2016). In this way, predators are often faced with the challenge of having their presence betrayed to potential prey by the chemical signals and cues they produce. Predator semiochemicals, like pheromones (e.g., sex attractants or territorial marking pheromones) and kairomones (e.g., metabolic biproducts), can persist in the environment for varying lengths of time, revealing the presence, identity, and abundance of emitting predators (Kats and Dill 1998; Dicke and Grostal 2001; Banks et al. 2016). Predators can also eavesdrop on chemical cues from other predators to assess prey availability and gauge possible competition (Stowe et al. 1995; Poelman et al. 2012; Mestre et al. 2014; Banks et al. 2016; Cusumano et al. 2020). Despite our current understanding of chemically mediated predator–prey interactions, we are lacking a systematic empirical evaluation of how chemical cues can be linked to species traits, like predator hunting mode, that affect predator and prey behavior. Evaluating these trophic interactions in a belowground soil environment, where chemical cues are the dominant type of communication between trophic levels, can help fill this knowledge gap.

Entomopathogenic nematodes (EPNs), in the genera *Steinernema* and *Heterorhabditis*, are important natural enemies of soil-dwelling insects and are emerging as model organisms for studies of belowground multi-trophic interactions (Campos-Herrera et al. 2012; Rasmann et al. 2012). Different species of EPNs exhibit a range of foraging modes, from cruisers that actively move through soil (e.g., *Heterorhabditis bacteriophora*) to sit-and-wait ambush predators (e.g., *Steinernema carpocapsae*) (Lewis et al. 2006; Griffin 2012; Ruan et al. 2018). EPNs are also associated with species-specific symbiotic bacteria that aid the free-living infective juveniles in infecting and killing their insect hosts (Ciche et al. 2006; Lewis et al. 2006). The insect-EPN-bacteria complex (i.e., infected host cadaver), produces a suite of chemical compounds including pheromones, insecticidal compounds, antimicrobials, and scavenging deterrents that influence EPN foraging behavior, infectivity, and survival (Hu et al. 1999; Hu and Webster 2000; Gulcu et al. 2012; Kaplan et al. 2012, 2020; Lu et al. 2017). Another recent discovery revealed EPN-infected insect cadavers emit olfactory cues that influence the behavior of their insect prey. These infected cadavers produce blends of volatile compounds distinct from the odors of dead insects, suggesting cadaver volatiles could reliably indicate increased predation risk to prey organisms (Helms et al. 2019; Zhang et al. 2019). Although some EPN olfactory cues may be conserved, there is emerging evidence for species-level specificity in their volatile blends and the corresponding insect responses (Helms et al. 2019; Zhang et al. 2019).

The goal of this study was to investigate how chemical cues from three entomopathogenic nematodes species, with different foraging strategies, influence the behavior of their insect herbivore prey and potential EPN competitors. First, we examined how cues from *Heterorhabditis bacteriophora* (cruiser EPNs)*, Steinernema riobrave* (intermediate EPNs), and *Steinernema carpocapsae* (ambusher EPNs) affect the foraging behavior of a root-feeding insect herbivore, striped cucumber beetle larvae (*Acalymma vittatum*). Based on previous studies, we predicted that beetle larvae would detect cues from EPN-infected cadavers as a warning of increased predation risk and avoid foraging near these cues, with the more sedentary *Steinernema* species eliciting the strongest avoidance response (Kats and Dill 1998; Luttbeg et al. 2020; Culshaw-Maurer et al. 2020). Because EPN-infected cadavers remain in soil near plant roots, we expected the concentrations of cadaver chemical cues to be similar for the three species, such that prey responses could be linked to blend differences. We then evaluated how foraging *Heterorhabditis bacteriophora* EPNs respond to cues produced by the three EPN species. Previous work indicates that cruiser EPNs rely on prey-associated cues while foraging (Grunseich et al. 2020b), and other research demonstrated that non-volatile pheromones from host cadavers affect dispersal in other EPN species (Oliveira-Hofman et al. 2019; Kaplan et al. 2020). We predicted that the EPNs would avoid cues from cadavers infected with the *Steinernema* species as a mechanism for avoiding interspecific competition. Finally, we characterized the volatile compounds produced by insect cadavers infected with each of the three EPN species to evaluate potential differences and conserved olfactory cues. We also investigated how EPN volatile blends change depending on insect host species, including wax moth larvae (*Galleria mellonella*), a standard rearing host for EPNs, and cucumber beetle larvae (*A. vittatum*), an ecologically relevant root-feeding herbivore. We predicted the EPN cues would vary by species, with the two more closely related *Steinernema* species producing more similar olfactory cues compared to the *Heterorhabditis* species, regardless of insect host species. By linking chemical cues to EPN species with different foraging strategies, we test the utility of the hunting mode hypothesis and examine how prey perceive predation risk and how predators recognize competition while foraging for critical resources. Our study suggests that EPN species identity and possibly their foraging strategies, have a significant context-dependent influence on belowground predator–prey and competitive interactions, calling attention to the cascading consequences ultimately shaping these ecological communities.

## Methods and Materials

### Nematodes, Insects, and Plants

The entomopathogenic nematode species used in this study (*Heterorhabditis bacteriophora*, *Steinernema riobrave,* and *Steinernema carpocapsae*) (Arbico Organics, Tucson, USA) are generalists with different foraging strategies that infect cucumber beetle larvae (*Acalymma vittatum*) (Ellers-Kirk et al. 2000). EPNs were cultured in last-instar wax moth larvae (*Galleria mellonella*) at 27 °C. Infective juveniles (IJs) were harvested in White traps. To generate EPN-infected insect cadavers for experiments, we added ~ 250 IJs to third-instar *A. vittatum* larvae or last-instar *G. mellonella* on moistened filter paper in 35 mm Petri dishes. Cadavers used in all experiments were 6 days post-infection for *G. mellonella* and 2 days post-infection for *A. vittatum* (approximately 2 days before IJ emergence). Control cadavers for all experiments were freeze killed and kept under the same conditions as EPN cadavers prior to experiments. Striped cucumber beetles (*A. vittatum*) were maintained in a laboratory colony on cultivated squash (*Cucurbita pepo* cv. Raven). Cucumber plants (*Cucumis sativus* cv. Max Pack) were grown from seed (Johnny's Selected Seeds, Fairfield, USA) and used in experiments after 3–4 weeks. Plants were grown in individual pots in topsoil mix (Hyponex Corporation, Marysville, USA) with 3 g Osmocote® fertilizer (Scotts, Marysville, USA) and kept in a growth room with supplemental lighting (16 h light: 8 h dark; 22 °C: 29 °C; 57% RH).

### Cucumber Beetle Responses to EPN Chemical Cues–Belowground Olfactometer

We conducted dual-choice experiments using belowground olfactometers to assess how chemical cues from EPN-infected insect cadavers influence the foraging behavior of cucumber beetle larvae. Two-choice olfactometers, consisting of two glass pots connected by a 13 cm-long glass arm with a central top opening were constructed. Individual cucumber seedlings were transplanted into the glass olfactometer pots in clean (baked at 200 °C for 24 h and cooled), moistened sand (10% water W/V), and allowed to acclimate for 24 h prior to experiments. Volatiles from the roots of these plants served as attractive cues for foraging beetle larvae (Grunseich et al. 2020b). For each trial, three EPN-infected cadavers were inserted at the base of one pot, while the other pot received three control cadavers. This was repeated for every EPN-insect species pair described above (beetle larvae with *H. bacteriophora* n = 9, *S. riobrave* n = 9, and *S. carpocapsae* n = 10; and wax moth larvae with *H. bacteriophora* n = 10, *S. riobrave* n = 10, and *S. carpocapsae* n = 12). To determine whether cucumber beetle larvae respond to cues from dead insects, we compared larval preference for plants with control cadavers vs. plants only for beetle (*A. vittatum*) or wax moth (*G. mellonella*) cadavers (n = 12). Olfactometers were assembled 30 min prior to experiments (no sand was present in the connecting arm (Robert et al. 2012)). We introduced 5 second-instar larvae into the central chamber and after 20 min, we recovered the larvae and recorded their locations.

### Cucumber Beetle Responses to *Heterorhabditis bacteriophora*-Infected Cadavers–Petri Dish Assays

To visually observe cucumber beetle behavioral responses to cues from *Heterorhabditis bacteriophora*-infected cadavers, we conducted Petri-dish preference assays (Fig. S1). On opposite sides of glass Petri dishes (15 mm × 100 mm), we placed three 5 cm segments of cucumber roots on moist filter paper. On one side, between the root segments, we placed *H. bacteriophora*-infected cadavers (3 *G. mellonella* or 5 *A. vittatum*). The other side received an equal number of control cadavers. Five second-instar beetle larvae were placed in the center and their locations and behavior (1. feeding on roots, 2. hiding, 3. feeding on cadavers, i.e., “cannibalism”, or 4. foraging/moving) were recorded after 10, 30, and 60 min (*A. vittatum*, n = 10*; G. mellonella*, n = 9). Preference was determined by location in the arena. Larvae that did not move from the center were recorded as “no-choice”.

### EPN (*Heterorhabditis bacteriophora*) Responses to EPN Chemical cues–Belowground Olfactometer

To investigate the influence of chemical cues from EPN-infected cadavers on the foraging behavior of *Heterorhabditis bacteriophora* EPN infective juveniles (IJs), we conducted two-choice preference assays with belowground olfactometers. *H. bacteriophora* use a “cruiser” foraging strategy and previous work indicates they are attracted to volatiles from beetle*-*damaged cucumber roots (Grunseich et al. 2020b). Two-choice belowground olfactometers, comprising two glass pots connected by a 36 cm-long glass arm with a central top opening were used in experiments. As above, cucumber seedlings were transplanted into olfactometer pots and EPN-infected cadavers or control cadavers were placed on each side. To induce production of EPN-attracting root volatiles, each plant was treated with 5 second-instar cucumber beetle larvae for 24 h. EPN IJs (2500) were added to the central chamber. After 48 h, sand was collected from each side of the olfactometer and IJs were extracted using an adapted Baermann funnel method and counted (Grunseich et al. 2020b). Larvae were recovered and confirmed to be feeding. This was repeated for both wax moths (*G. mellonella*) and beetles (*A. vittatum*) infected with each of the three EPN species (*H. bacteriophora*, *S. riobrave*, and *S. carpocapsae*; n = 6).

### Collection and Analysis of EPN Volatiles

To evaluate potential differences among EPN-produced olfactory cues, we characterized the volatiles emitted by the three species of EPNs, each infecting two insect species (beetle larvae *A. vittatum* with *H. bacteriophora* n = 14*, S. riobrave* n = 10*,* and *S. carpocapsae* n = 9; and wax moth larvae *G. mellonella* with each species n = 10). As controls, we analyzed volatiles produced by freeze-killed *A. vittatum* (n = 17) and *G. mellonella* (n = 10) cadavers. We used solid-phase microextraction (SPME) to collect volatiles from the headspace of each cadaver treatment (Zhang et al. 2019; Fu et al. 2020). Individual cadavers were placed into 4 ml glass vials with a PTFE septum-containing lid. Vials were held at 35 °C for 1 h, then a SPME fiber (100 µm, polydimethylsiloxane, Agilent Technologies, Palo Alto, USA) was inserted and exposed for 1 h for *G. mellonella* or 2 h for *A. vittatum* cadavers (statistical comparisons were not made between the two insect host species as sampling conditions differed). Samples were analyzed using an Agilent 7890B gas chromatograph and 5977B mass spectrometer with a splitless injector held at 250 °C and helium as the carrier gas. The column (HP-5MS 30 m × 0.250 mm-ID, 0.25 μm film thickness, Agilent Technologies, Palo Alto, USA) was held at 60 °C for 1 min then increased at 5 °C min^−1^ until 200 °C. Compounds were ionized by electron impact ionization at 70 eV and mass spectra were acquired by scanning from 40 to 300 m/z at 5.30 scans s^−1^. Tentative identification of target compounds was achieved by comparison with mass spectral libraries (NIST17, Adams2 (Allured Publishing Corporation)), and structure assignments were confirmed where possible by comparisons of mass spectra and retention times with authentic standards. Compounds are reported as peak area per cadaver mass.

### Statistical analyses

Statistical analyses were conducted in the software program R (R Version 3.6.3, R Development Core Team, 2020). Preference data were analyzed using generalized log-linear models (GLM) with quasi-likelihood functions to compensate for overdispersion (Ali et al. 2010). Non-metric multidimensional scaling (NMDS) ordinations were used to visualize volatile blend differences (package vegan, Oksanen et al., 2012). Spiders connect all points within a treatment and ellipses show the standard deviation around centroids. Permutational multivariate analysis of variance (PERMANOVA) was conducted to assess differences among cadaver odor blends for each EPN species (insect host species compared separately).

## Results

### Cucumber Beetle Larvae Avoid Chemical Cues from *Heterorhabditis bacteriophora*-Infected Cadavers

**Fig. 1:**
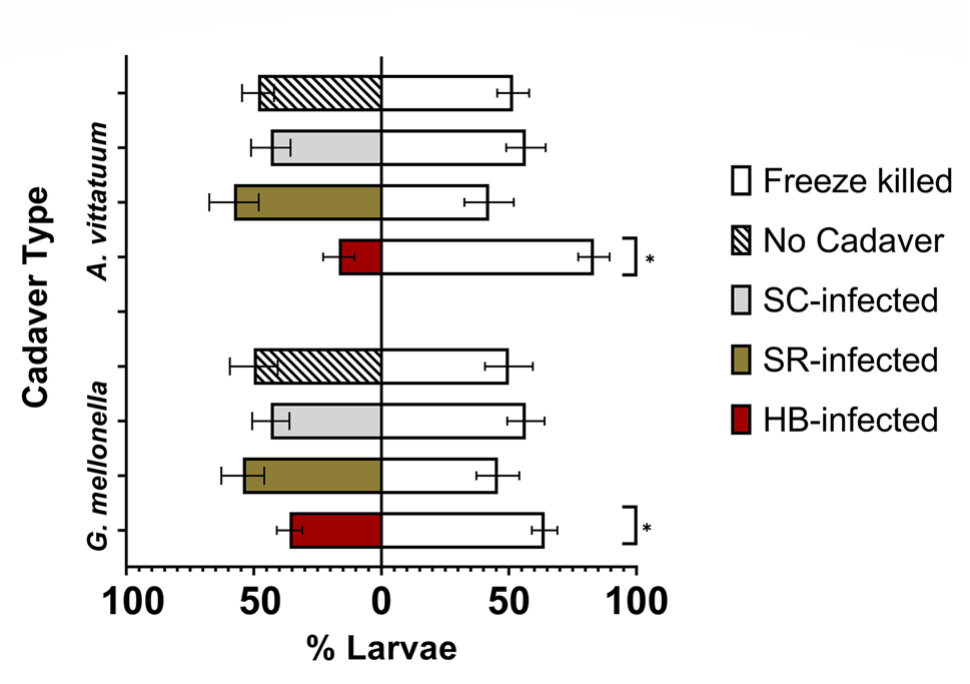
Cucumber beetle larvae avoided plants with cues from *Heterorhabditis bacteriophora-*infected cadavers. Larval preference was not influenced by cues from *Steinernema carpocapsae-* or *Steinernema riobrave-*infected cadavers or control cadavers. Means ± SE are presented. (*p ≤ 0.05)

Foraging cucumber beetle larvae differentiated between chemical cues from *Heterorhabditis bacteriophora-*infected and uninfected control cadavers, avoiding the cruiser EPN-infected cadaver cues. This was true for both beetle (*A. vittatum*) cadavers (*GLM* *T*_1,8_ = 10.96, *p* < 0.001) and wax moth (*G. mellonella*) cadavers (*GLM T*_1,9_ =  − 3.96, *p* < 0.001). Contrary to our predictions, however, beetle larvae did not avoid cues from the other two EPN species, regardless of insect host species (Fig. 1). Larvae did not differentiate between *A. vittatum* infected with *Steinernema riobrave* or control cadavers (*GLM* *T*_1,8_ = -1.136, *p* = 0.27) or *S. riobrave*-infected *G. mellonella* vs. controls (*GLM T*_1,8_ = 1.30, *p* = 0.22). They also failed to differentiate *Steinernema Carpocapsae*-infected *A. vittatum* or control cadavers (*GLM T*_1,9_ = 1.22, *p* = 0.24) and *S. Carpocapsae*-infected *G. mellonella* or controls (*GLM T*_1,11_ = 1.29, *p* = 0.21) (Fig. 1). Cues from freeze-killed cadavers did not influence beetle foraging compared to plants only for *A. vittatum* cadavers (*GLM* *T*_1,11_ = 0.377, *p* = 0.71) or *G. mellonella* cadavers (*GLM* *T*_1,11_ = 0.01, *p* = 1.00) (Fig. 1).

### Cucumber Beetle Larvae Avoid Insect Cadavers Infected with *Heterorhabditis bacteriophora* EPNs

**Fig. 2:**
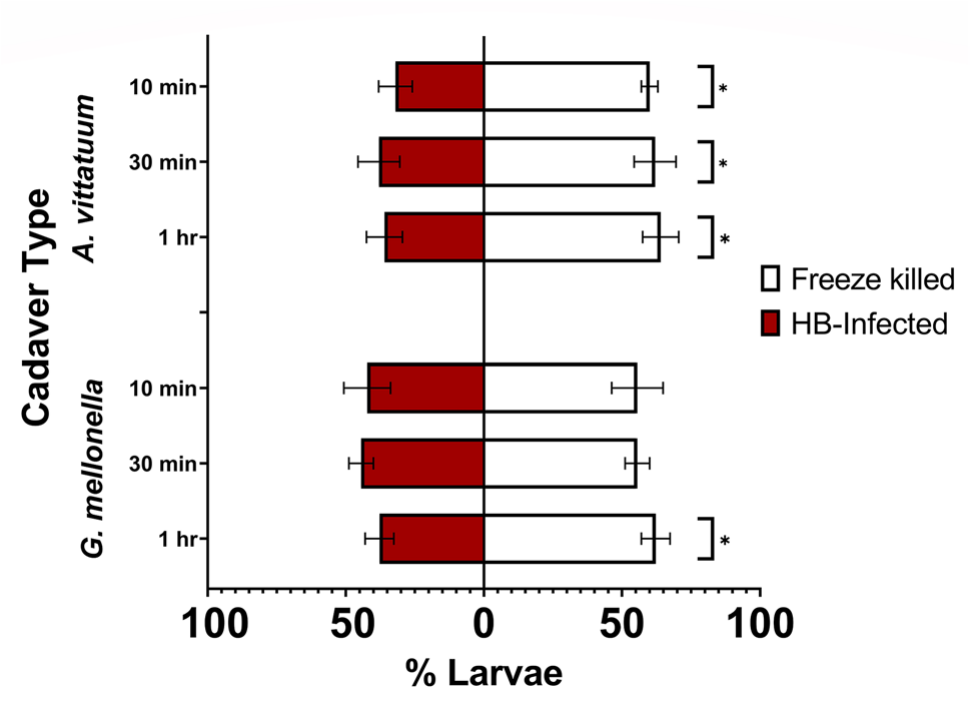
Cucumber beetle larvae avoided conspecific (*A. vittatum*) and heterospecific (*G. mellonella*) cadavers infected with *Heterorhabditis bacteriophora*. Means ± SE are presented. (*p ≤ 0.05)

In Petri-dish preference assays, we observed a similar EPN avoidance response by *A. vittatum* beetle larvae. More larvae avoided *Heterorhabditis bacteriophora*-infected conspecifics and nearby roots throughout the duration of the experiment (10 min *GLM T*_1,18_ = -4.11, *p* < 0.001, 30 min *GLM T*_1,18_ = -2.24, *p* = 0.037, and 1 h *GLM* *T*_1,18_ = -2.70, *p* = 0.014) (Fig. 2). In addition to feeding on roots, cucumber beetle larvae—which readily cannibalize conspecifics when food is limited—also consumed uninfected conspecific cadavers, but not EPN-infected cadavers. In total, we observed “cannibalism” of 40.74% of freeze-killed larvae. Foraging larvae did not discriminate between *Heterorhabditis bacteriophora*-infected and control wax moth (*G. mellonella*) cadavers until after 1 h of foraging (10 min *GLM T*_1,17_ = 1.06, *p* = 0.30, 30 min *GLM T*_1,17_ = 1.76, *p* = 0.096, 1 h *GLM* *T*_1,17_ = -3.31, *p* = 0.004) (Fig. 2). No *G. mellonella* cadavers were consumed by the beetle larvae.

### *Heterorhabditis bacteriophora* EPN IJs are Attracted to Chemical Cues from *Steinernema*-Infected Insect Cadavers

**Fig. 3:**
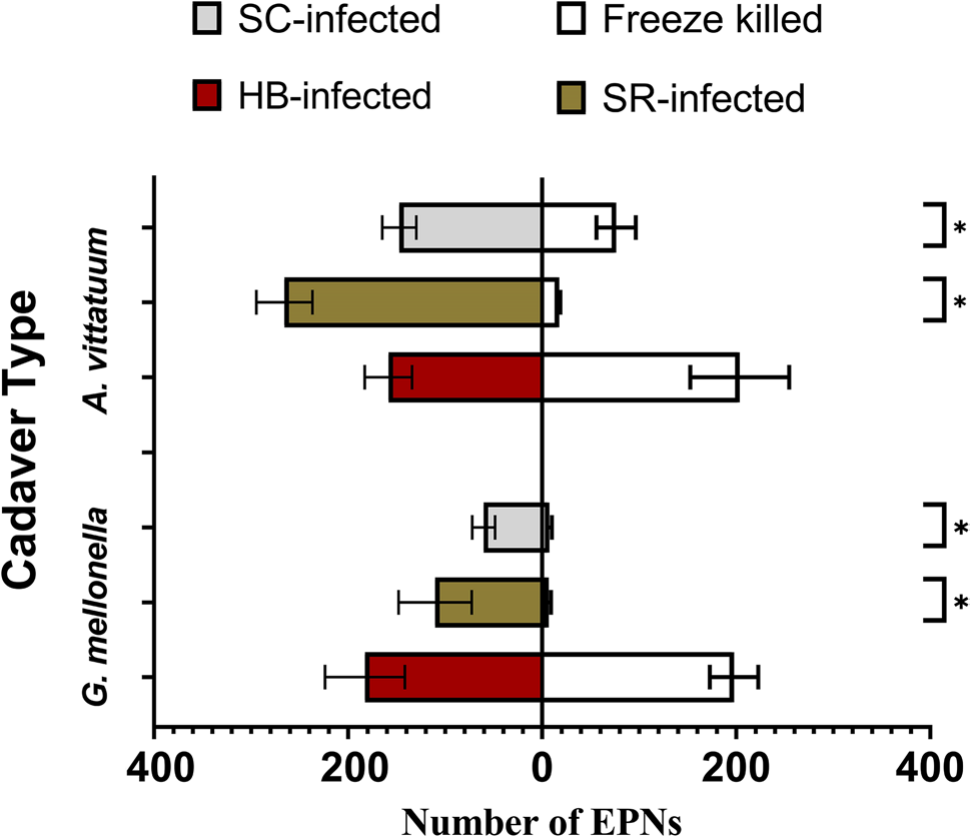
*Heterorhabditis bacteriophora* EPN IJs preferred chemical cues from *Steinernema riobrave-* and *Steinernema carpocapsae*-infected cadavers, while cues from *H. bacteriophora*-infected cadavers did not affect conspecific foraging. (*p ≤ 0.05). Means ± SE are presented

Contrary to our predictions, we found that *Heterorhabditis bacteriophora* EPN IJs were attracted to cues from *Steinernema carpocapsae*-infected *G. mellonella* cadavers (*GLM T*_1,5_ = 3.98, *p* = 0.003) and *S. carpocapsae-*infected *A. vittatum* cadavers (*GLM T*_1,5_ = 2.65, *p* = 0.029). They were also attracted to *Steinernema riobrave-*infected cadaver cues regardless of host species (*G. mellonella, GLM T*_1,5_ = 2.736, *p* = 0.025; *A. vittatum, GLM T*_1,5_ = 8.57, *p* < 0.001) (Fig. 3). In contrast, *Heterorhabditis bacteriophora* EPN IJs did not differentiate between cues from conspecific-infected cadavers and freeze-killed control cadavers (*G. mellonella, GLM T*_1,5_ = -0.32, *p* = 0.76; *A. vittatum, GLM T*_1,5_ = 0.125, *p* = 0.903) (Fig. 3).

### EPN Olfactory Cues Vary Across EPN Species and by Insect Host Species

**Fig. 4:**
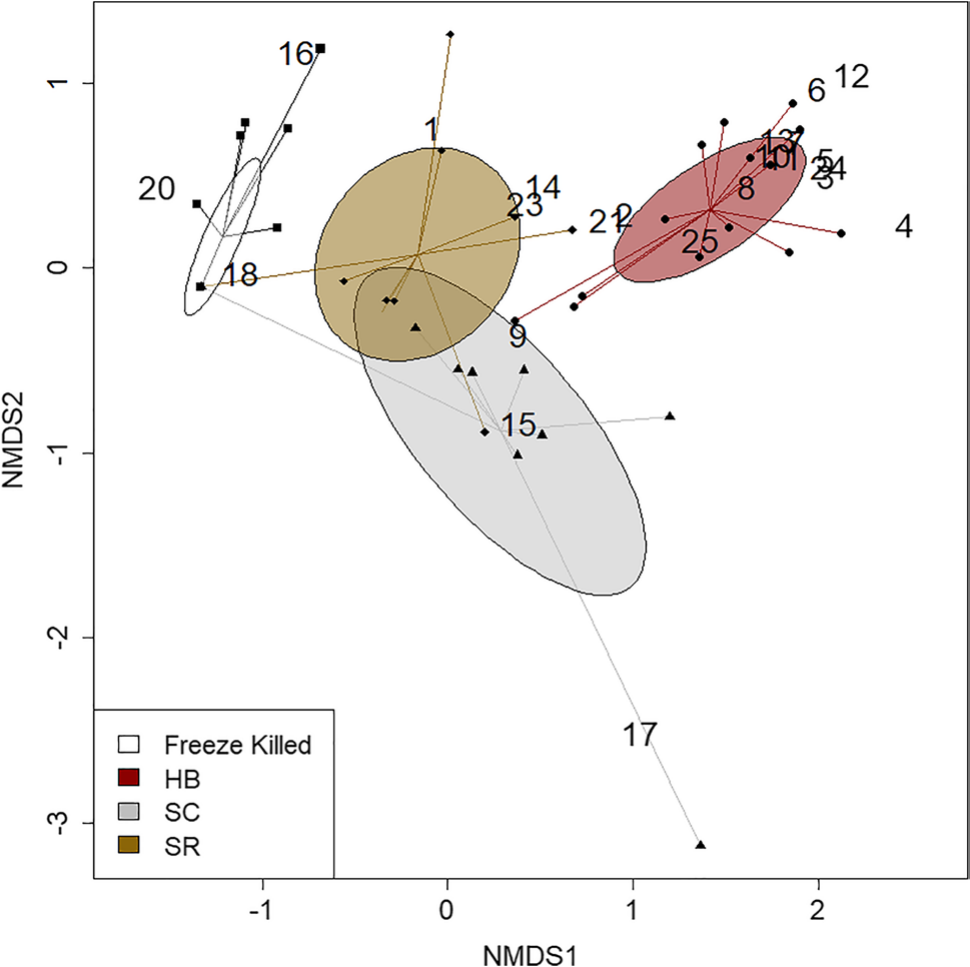
Different volatile blends were emitted by *A. vittatum* beetle cadavers infected with three EPN species and freeze-killed controls. FK = Freeze-killed cadaver; HB = *Heterorhabditis bacteriophora*; SC = *Steinernema carpocapsae*; SR = *Steinernema riobrave.* Numbered compounds are listed in Table 2

**Fig. 5:**
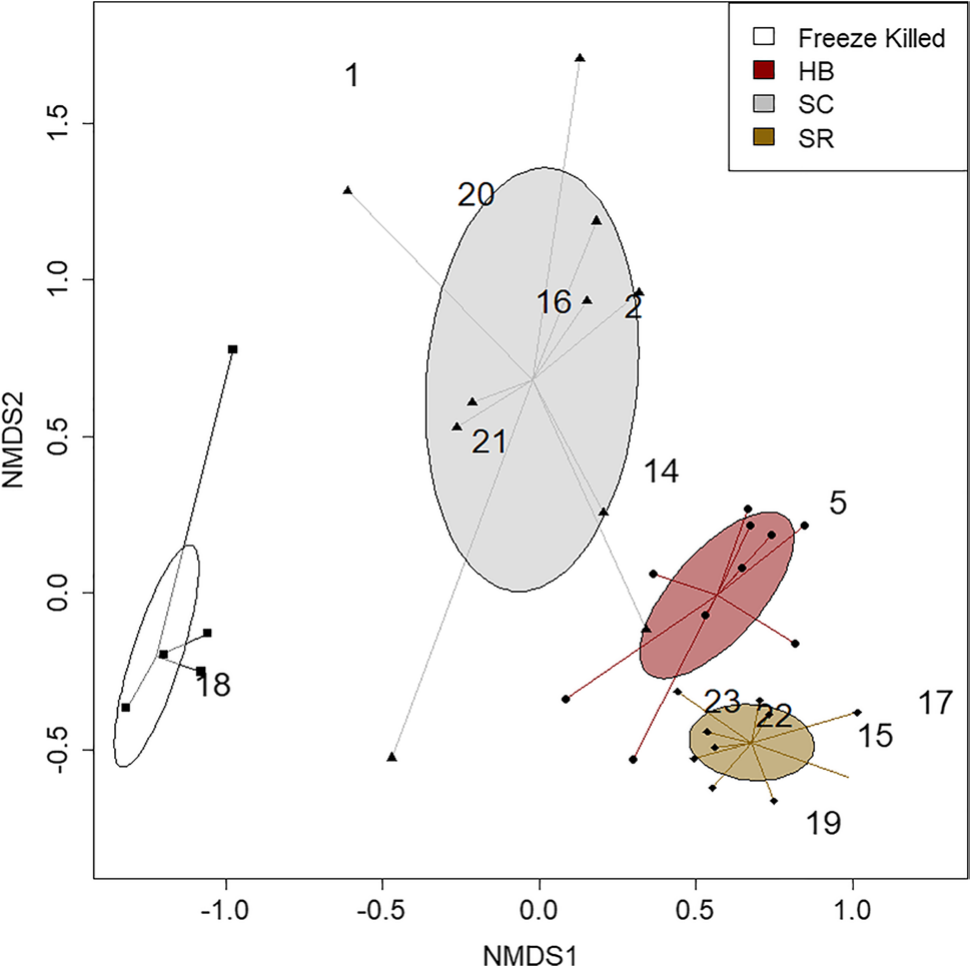
Distinct volatile blends were emitted by wax moth larvae (*G. mellonella*) cadavers infected with 3 species of EPNs and freeze-killed controls. FK = Freeze-killed cadaver; HB = *Heterorhabditis bacteriophora*; SC = *Steinernema carpocapsae*; SR = *Steinernema riobrave.* Numbered compounds are listed in Table 2

We identified differences in the volatile blends from cucumber beetle (*A. vittatum*) and wax moth larvae (*G. mellonella*) cadavers infected with the 3 EPN species (Table 1, Fig. 4, 5). We recovered 25 volatile compounds across all treatments, with qualitative and quantitative differences in the species blends (Table 2). Notably, we found a suite of seven sesquiterpenes that were only emitted by *Heterorhabditis bacteriophora-*infected beetle cadavers. These were tentatively identified (from their mass spectral data) as α-copaene, β-cubebene, **γ**-cadinene, δ-cadinene, β-copaene, **γ**-muurolene, and δ-amorphene (Table 2). The compound 1-dodecene was only present for cadavers infected with *Heterorhabditis bacteriophora* for both insect host species (Table 2). Butylated hydroxytoluene and unknown 6 were emitted by all EPN-infected *A. vittatum* beetle cadavers, but not by control cadavers or any *G. mellonella* cadavers (Table 2). The compound 1-nonene was emitted by cadavers infected with each of the 3 EPN species (Table 2). When comparing the overall volatile blends of *A. vittatum* beetle cadavers, we found that the two *Steinernema* species were more similar to each other than *Heterorhabditis* and that all three were different from the freeze-killed controls (Table 1, Fig. 4). The differences among volatile blends from *G. mellonella* cadavers were more pronounced, with little similarity between any EPN species (Table 1, Fig. 5).

**Table 1:** Results (*p-*values) from individual PERMANOVA comparisons across all EPN treatments for each insect host species

Permutation ANOVA *P*-values							
Cadaver	Gmel HB	Gmel SC	Gmel SR	Cadaver	Avit HB	Avit SC	Avit SR
Gmel FK	< 0.001	< 0.001	< 0.001	Avit FK	< 0.001	< 0.001	< 0.001
Gmel HB		0.002	< 0.001	Avit HB		0.001	< 0.001
Gmel SC			< 0.001	Avit SC			0.024

Gmel = *G. mellonella*; Avit = *A. vittatum*; FK = Freeze-killed cadaver; HB = *H. bacteriophora*; SC = *S. carpocapsae*; SR = *S. riobrave*

**Table 2:** Individual compounds from *G. mellonella* and *A. vittatum* cadavers infected with *Heterorhabditis bacteriophora*, *Steinernema riobrave*, or *Steinernema carpocapsae* or uninfected controls

	Cadaver Types				
Compound	Insect Host Species	Control ± SE	HB ± SE	SR ± SE	SC ± SE
benzaldehyde (1)	*A. vittatum*	4041.34 ± 2308.8	78,957.50 ± 52,835.8	2591.74 ± 2591.7	n.d
	*G. mellonella*	n.d	8.03 ± 6.0	n.d	301.63 ± 301.6
1-nonene (2)	*A. vittatum*	n.d	4922.81 ± 2695.7	69,106.05 ± 67,229.2	2495.74 ± 2495.7
	*G. mellonella*	n.d	292.47 ± 76.5	2.64 ± 2.6	502.98 ± 201.8
1-decene (3)	*A. vittatum*	n.d	22,981.4 ± 8843.1	n.d	n.d
	*G. mellonella*	n.d	n.d	n.d	n.d
5-decene (4)	*A. vittatum*	n.d	2364.19 ± 2364.2	n.d	n.d
	*G. mellonella*	n.d	n.d	n.d	n.d
1-dodecene (5)	*A. vittatum*	n.d	59,284.39 ± 24,186.6	n.d	n.d
	*G. mellonella*	n.d	19.20 ± 7.12	n.d	n.d
α-copaene (6)	*A. vittatum*	n.d	6324.53 ± 5140.6	n.d	n.d
	*G. mellonella*	n.d	n.d	n.d	n.d
β-cubebene (7)	*A. vittatum*	n.d	4604.18 ± 2953.3	n.d	n.d
	*G. mellonella*	n.d	n.d	n.d	n.d
γ-cadinene (8)	*A. vittatum*	n.d	279,229.35 ± 202,155.5	n.d	n.d
	*G. mellonella*	n.d	n.d	n.d	n.d
butylated hydroxytoluene (9)	*A. vittatum*	n.d	194,954.80 ± 65,435.3	192,631.60 ± 67,907.8	319,336.43 ± 82,041.5
	*G. mellonella*	n.d	n.d	n.d	n.d
δ-cadinene (10)	*A. vittatum*	n.d	6178.03 ± 2835.4	n.d	n.d
	*G. mellonella*	n.d	n.d	n.d	n.d
β-copaene (11)	*A. vittatum*	n.d	5576.12171 ± 2903.6	n.d	n.d
	*G. mellonella*	n.d	n.d	n.d	n.d
γ-muurolene (12)	*A. vittatum*	n.d	1252.35 ± 1109.9	n.d	n.d
	*G. mellonella*	n.d	n.d	n.d	n.d
δ-amorphene (13)	*A. vittatum*	n.d	5391.73 ± 2842.7	n.d	n.d
	*G. mellonella*	n.d	n.d	n.d	n.d
indole (14)	*A. vittatum*	n.d	5273.03 ± 1810.3	83,654.50 ± 73,158.6	17,637.17 ± 14,693.9
	*G. mellonella*	37.18 ± 19.8	5993.10 ± 1958.8	338.76 ± 108.8	8271.36 ± 4636.1
2,5-dimethyl-pyrazine (15)	*A. vittatum*	389.85 ± 389.9	n.d	10,182.12 ± 10,182.1	484,459.47 ± 249,474.9
	*G. mellonella*	n.d	1024.07 ± 1024.1	374.16 ± 157.1	n.d
phenol (16)	*A. vittatum*	9894.87 ± 6873.4	n.d	28,993.37 ± 26,597.1	n.d
	*G. mellonella*	25.93 ± 17.3	2.07 ± 2.0	648.36 ± 232.8	4453.59 ± 1980.6
trimethyl-pyrazine (17)	*A. vittatum*	n.d	n.d	8534.07 ± 8534.0	9361.00 ± 3895.6
	*G. mellonella*	n.d	13.20 ± 13.2	17.29 ± 12.0	n.d
phenyl ethyl alcohol (18)	*A. vittatum*	60,529.06 ± 21,765.4	7012.90 ± 1439.2	58,051.81 ± 13,100.4	12,493.10 ± 4582.8
	*G. mellonella*	522.86 ± 203.3	249.91 ± 42.0	126.70 ± 15.0	118.332 ± 25.1
anisole (19)	*A. vittatum*	n.d	n.d	n.d	n.d
	*G. mellonella*	n.d	n.d	1882.12 ± 367.7	n.d
unknown 1 (20)	*A. vittatum*	35,731.40 ± 35,731.4	n.d	n.d	n.d
	*G. mellonella*	460.48 ± 460.4	n.d	12.04 ± 8.6	262.29 ± 146.3
unknown 2 (21)	*A. vittatum*	n.d	149.26 ± 149.2	36,590.80 ± 36,590.8	n.d
	*G. mellonella*	192.01 ± 192.0	n.d	66.00 ± 30.0	40.12 ± 40.1
unknown 3 (22)	*A. vittatum*	n.d	n.d	n.d	n.d
	*G. mellonella*	n.d	247.20 ± 30.0	357.07 ± 56.7	13.67 ± 10.6
unknown 4 (23)	*A. vittatum*	n.d	n.d	1466.21 ± 1466.2	n.d
	*G. mellonella*	n.d	1559.06 ± 207.4	2315.6 ± 356.2	183.39 ± 100.1
unknown 5 (24)	*A. vittatum*	n.d	38,297.77 ± 15,397.4	n.d	n.d
	*G. mellonella*	n.d	n.d	n.d	n.d
unknown 6 (25)	*A. vittatum*	n.d	120,966.59 ± 35,523.5	13,208.94 ± 12,005.8	7832.31 ± 7832.3
	*G. mellonella*	n.d	n.d	n.d	n.d

n.d. = not detected. Means ± SE are presented

## Discussion

The outcomes of trophic interactions are often affected by traits of the interacting species, with predator traits driving responses in both prey and competitors. However, our understanding of these traits and how they vary across natural enemy species with different hunting modes, particularly in belowground soil environments, remains limited. Here, we found that EPN chemical cues varied across three species with different foraging strategies. Cues from active-foraging *Heterorhabditis bacteriophora* EPNs repelled foraging prey, while cues from the ambusher and intermediate-foraging *Steinernema* EPNs did not affect prey behavior. Further, active-foraging cruiser EPNs were attracted to heterospecific cues but showed no response to conspecific cues. Taken together, our findings indicate chemical cues from EPNs play an integral role in shaping belowground predator–prey and competitive interactions, highlighting the context dependency of chemically mediated trophic interactions, and raising additional questions about how organisms interpret the information provided by predator-associated cues.

### Cucumber Beetle Larvae Respond Differently to Chemical Cues from Different EPN Species

In foraging for food resources, prey must simultaneously avoid predation (Sih 1980) and many do so by adaptively responding to chemical cues associated with a heightened risk of predation. Our previous work suggests that *A. vittatum* beetle larvae are “risk averse” and repelled by olfactory cues from herbivore-damaged plants, presumably because these cues also attract EPN natural enemies (Grunseich et al. 2020b). Here, we evaluated whether larvae can also reduce their predation risk by avoiding chemical cues produced directly by EPNs. Cucumber beetle larvae likely rely on avoidance or escape behavior as the first level of defense against EPNs, which agrees with our findings that larvae were repelled by some EPN chemical cues (Fig. 1). Notably, in our Petri dish assays, we also observed “cannibalism” of uninfected control cadavers, while larvae avoided the EPN-infected individuals. This agrees with our previous observations that in the absence of adequate food resources, *A. vittatum* larvae readily cannibalize conspecifics, and provides further evidence that they avoid EPN-associated cues. Previous studies have also reported elevated incidence of cannibalism among prey exposed to increased predation risk, likely as a mechanism to enhance performance (Tigreros et al. 2017). In our study, the presence of EPN cues could have triggered a fear-induced cannibalism response among the larvae.

Several recent studies have focused on how predator traits, including hunting modes, influence the outcomes of predator–prey interactions (Preisser et al. 2007; Pears et al. 2018; Luttbeg et al. 2020). Current hypotheses related to prey perception of predation risk suggest prey should respond most strongly to cues from sedentary predators, as these cues may be more concentrated and indicate a more immediate threat compared to an active predator who is more likely to vacate a shared microhabitat relatively quickly (Kats and Dill 1998; Preisser et al. 2007; Schmitz et al. 2008; Kuijper et al. 2015). Contrary to these predictions, we found that cucumber beetle larvae avoided chemical cues from the active-hunting species, *Heterorhabditis bacteriophora*, and did not respond to cues from the other two, more sedentary EPN species (Fig. 1, 2). Cadavers in experiments were standardized for age and size rendering it unlikely that our results are due to cue intensity. An alternative to the predator hunting mode hypothesis suggests prey should detect and avoid the most lethal predators. Although, all three EPN species kill *A. vittatum* larvae, future studies should examine whether *Heterorhabditis bacteriophora* pose a greater infection risk than the *Steinernema* species. Previous research suggests *Steinernema carpocapsae* is less effective for controlling belowground root-feeding herbivores compared to *Heterorhabditis bacteriophora*, however, this is due to differences in host-finding and not infection ability (Toepfer et al. 2005; Lortkipanidze et al. 2016)*.*

Another possible explanation for why cucumber beetle larvae avoided *Heterorhabditis bacteriophora-*infected cadavers, but not the other EPN species, is that these more sedentary *Steinernema* species face strong selection against production of chemical cues that would repel their prey. This type of chemical crypsis has been predicted but little evidence has been identified to date (Kats and Dill 1998; Ruxton 2009; Miller et al. 2015). The specific EPN cues responsible for repelling *A. vittatum* beetle larvae and their roles in EPN ecology, as well as the potential for EPN olfactory crypsis, merit further investigation. Further, because the *Steinernema*-associated cues and corresponding beetle responses were more similar, it is also possible these results were driven by EPN taxonomic relatedness. Future work should expand on these efforts by including additional EPN species from both genera.

### Foraging *Heterorhabditis bacteriophora* EPN IJs are Attracted to Chemical Cues from Heterospecific EPN-Infected Cadavers

Many species of EPNs use chemical cues, often emitted by damaged plant roots, to locate their insect herbivore hosts (Grewal et al. 1997; Rasmann et al. 2005; Ali et al. 2010), this includes “cruisers” like *Heterorhabditis bacteriophora* (Grunseich et al. 2020b). Here we tested whether foraging *H. bacteriophora* EPN IJs respond to chemical cues from conspecific or heterospecific EPN-infected cadavers. Previous studies of *Steinernema* sp. have yielded contrasting results, suggesting that some but not all EPN species use cadaver cues to avoid interspecific competition (Grewal et al. 1997; Fu et al. 2020). We predicted that foraging *Heterorhabditis bacteriophora* would avoid chemical cues from other EPN species to bypass competition. However, we instead found they were attracted to heterospecific cues and did not respond to cues from conspecifics when these were presented with attractive *C. sativus* root volatiles. This suggests that either heterospecific cadaver cues alone or combined cadaver and root cues could indicate prey availability to *Heterorhabditis bacteriophora* and that this response overrides avoidance of interspecific competition. It is also possible that *Heterorhabditis bacteriophora* is a superior competitor against the *Steinernema* sp. used in this study. Previous reports indicate that *H. bacteriophora* cannot reproduce as scavengers in freeze-killed *G. mellonella* (Blanco-Pérez et al. 2019), but that their performance is positively affected by co-infection with *Steinernema carpocapsae* and *Steinernema feltiae* (Neumann and Shields 2006), lending further support to this idea. Co-existence between different EPN species is possible, particularly if prey resources are abundant and predators separate into different spatial niches, for example along vertical gradients (Kaya and Koppenhöfer 1996; Ram et al. 2008).

### Olfactory Cues from EPN-Infected Cadavers are Species Specific

A growing number of studies provide evidence that predators produce specific chemical cues, that are detected by both prey and competitors (Gonthier 2012; Siepielski et al. 2016; Banks et al. 2016). Here, we focused on olfactory cues from insect cadavers infected with EPNs, which represent a unique class of predator-associated semiochemicals, combining necromones from the dead insect host with predator kairomones (Helms et al. 2019; Zhang et al. 2019). This aligns with our findings that EPN-infected cadavers emit different blends of volatile compounds compared to dead and decomposing insects (Fig. 4, 5), and suggests they could provide a reliable indicator of EPN presence to susceptible insect prey or other competing predators.

A surprising finding in this study was that the three EPN species produced distinct blends of olfactory cues (Fig. 4, 5), with little overlap across the various EPN-host species combinations (Table 2). Although species-level differences have been implicated from previous work (Helms et al. 2019; Zhang et al. 2019; Fu et al. 2020), we expected to find a suite of conserved cues associated with EPN infection. However, only the compound 1-nonene was present for all EPN species combinations. Even the two *Steinernema* species, which we predicted would be more similar compared to *Heterorhabditis*, produced distinct volatile blends with relatively little compound overlap (Fig. 4, 5). Previous studies have documented other conserved EPN semiochemicals, including their ascaroside pheromones, which appear to be chemically similar across EPN and even plant-parasitic nematode species (Choe et al. 2012). This begs the question “why are EPN-produced volatiles so different among species?”. One possible explanation stems from the highly specific associations of different EPN species with different species of bacterial symbionts. *Steinernema* are known to form associations with *Xenorhabdus* sp. (e.g. *S. carpocapsae* with *X. nematophila* and *S. riobrave* with *X. cabanillasii*), while *Heterorhabditis* associate with *Photorhabdus* sp. (e.g. *H. bacteriophora* with *P. luminescens*) (Lewis et al. 2006; Campos-Herrera et al. 2012). These bacteria play critical roles in host infection, deterring other microorganisms or scavengers, and even mediating interspecific competition, often through synthesizing bioactive chemicals (Sicard et al. 2006; Cai et al. 2017; Machado et al. 2020). It is possible these different symbiont species are at least partially responsible for driving the high degree of interspecific variation among EPN volatile blends.

Another unexpected result was the dramatic difference in cadaver volatile blends from the two insect host species infected with the same species of EPNs. Remarkably, cucumber beetle (*A. vittatum*) cadavers infected with *Heterorhabditis bacteriophora,* but not wax moth (*G. mellonella*) cadavers, produced sesquiterpenes as part of their volatile blends (Table 2). These compounds are not produced by *A. vittatum* alone or their host plant, and to our knowledge, this is the first report of terpene production from EPNs and/or their symbionts (Helms et al. 2019; Zhang et al. 2019). Such differences may stem from EPN symbionts aiding in the breakdown of host nutrients and secondary metabolites, as microbes grown on different substrates can change microbial metabolite profiles (Borjesson et al. 1990; Davis et al. 2013). Further research is required to tease apart the contributions of EPN microbial symbionts to cadaver volatile blends.

## Supplementary Information

Below is the link to the electronic supplementary material.

Supplementary file1 (PDF 157 kb)

## Data Availability

Data will be available from the Dryad Digital Repository following acceptance for publication.
