# Supplementary material for: Chemical Cues from Entomopathogenic Nematodes Vary Across Three Species with Different Foraging Strategies, Triggering Different Behavioral Responses in Prey and Competitors
Source: J Chem Ecol. 2021 Aug 20;47(10-11):822–33. doi: 10.1007/s10886-021-01304-8 (PMC8613145; doi:10.1007/s10886-021-01304-8)

Figure S1-A – Petri Dish Preference Assay (*G. mellonella)*

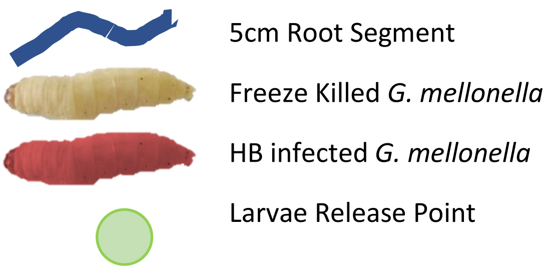

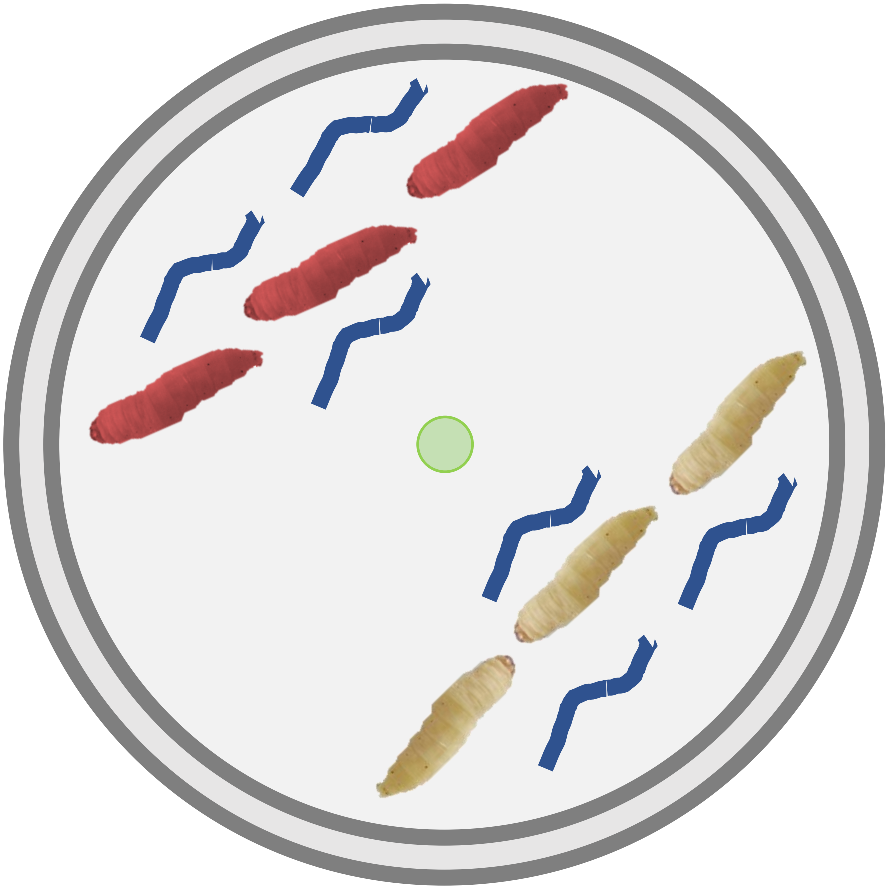

Figure S1-B – Petri Dish Preference Assay (*A. vittatum)*

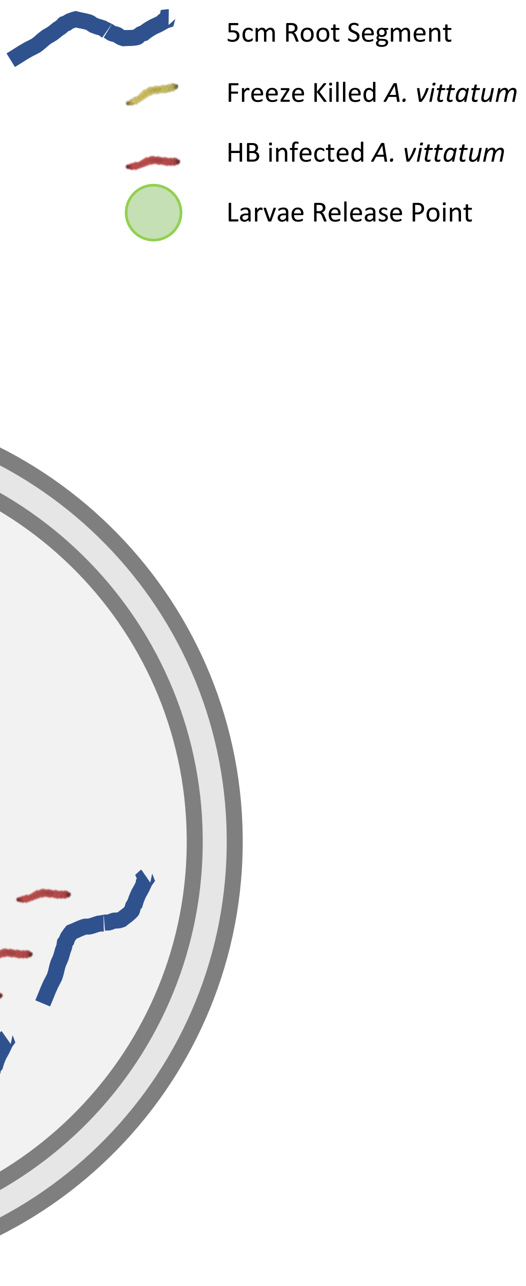

Supplement: Supplementary file 1 — Supplementary file1 (PDF 157 kb) [file 10886_2021_1304_MOESM1_ESM.pdf]
